# HIV Care Cascade Among Adolescents in a “Test and Treat” Community-Based Intervention: HPTN 071 (PopART) for Youth Study

**DOI:** 10.1016/j.jadohealth.2020.07.029

**Published:** 2021-04

**Authors:** Kwame Shanaube, David Macleod, Mwate Joseph Chaila, Constance Mackworth-Young, Graeme Hoddinott, Ab Schaap, Sian Floyd, Peter Bock, Richard Hayes, Sarah Fidler, Helen Ayles

**Affiliations:** a Zambart, Lusaka, Zambia; b Department of Infectious Disease Epidemiology, London School of Hygiene and Tropical Medicine, London, United Kingdom; c Ministry of Health, Lusaka, Zambia; d Department of Global Health and Development, London School of Hygiene and Tropical Medicine, London, United Kingdom; e Desmond Tutu TB Centre, Department of Pediatrics and Child Health, Faculty of Medicine and Health Sciences, Stellenbosch University, Cape Town, South Africa; f Faculty of Medicine, Department of Infectious Disease, Imperial College, London, United Kingdom; g Department of Clinical Research, London School of Hygiene and Tropical Medicine, London, United Kingdom

**Keywords:** Adolescents, HIV care cascade, 90-90-90 targets, Combination HIV prevention, Sub-Saharan Africa

## Abstract

**Purpose:**

The PopART for Youth (P-ART-Y) study was nested within the HPTN 071 (PopART) trial, a three-arm community randomized trial in 21 communities in Zambia and South Africa. The P-ART-Y study evaluated the acceptability and uptake of a combination HIV prevention package among young people. We report on the HIV care cascade for adolescents aged 10–19 years from 14 communities receiving the full HIV prevention package in Zambia and South Africa.

**Methods:**

Adolescents were offered participation in the PopART intervention, which included universal home-based HIV testing, linkage to care, antiretroviral therapy (ART) adherence, and other services. Data were collected from September 2016 to December 2017, covering the third round (R3) of the intervention.

**Results:**

We enumerated (listed) 128,241 adolescents (Zambia: 95,295 and South Africa: 32,946). Of the adolescents offered HIV testing, 81.9% accepted in Zambia and 70.3% in South Africa. Knowledge of HIV status was higher among older adolescents and increased from 31.4% before R3 to 88.3% at the end of R3 in Zambia and from 28.3% to 79.5% in South Africa. Overall, there were 1,710 (1.9%) adolescents identified as living with HIV by the end of R3 (515 new diagnoses and 1,195 self-reported). Of the new diagnoses, 335 (65.0%) were girls aged 15–19 years. The median time to initiate ART was 5 months. ART coverage before and after R3 increased from 61.3% to 78.7% in Zambia and from 65.6% to 87.8% in South Africa, with boys having higher uptake than girls in both countries.

**Conclusions:**

The PopART intervention substantially increased coverage toward the first and second UNAIDS 90-90-90 targets in adolescents.

**Implications and Contribution**

Limited data exist on the HIV care cascade among adolescents living in Sub-Saharan Africa. A community-based universal combination HIV prevention package substantially increased coverage toward the first and second UNAIDS 90-90-90 targets in adolescents aged 10–19 years, although gaps remain in some age/sex groups.

HIV/AIDS is the second most frequent cause of death for adolescents aged 10–19 years globally and the leading cause of death in Sub-Saharan Africa (SSA) [1]. Adolescent deaths are largely due to attrition throughout the HIV care cascade: low knowledge of HIV status, delayed linkage to care (LTC) and uptake of antiretroviral therapy (ART), low retention and adherence to ART, and, therefore, poor viral suppression [2].

The “HIV care cascade,” also known as the HIV care continuum, outlines the sequential steps of HIV care from the initial diagnosis to the goal of viral suppression [3,4]. When compared with adults, evidence suggests that adolescents living with HIV (ALHIV) are less likely to know their HIV status, are more likely to be lost to follow-up after registering for HIV care, have suboptimal adherence to ART, experience higher rates of virologic failure, and have worse outcomes across the cascade [5].

With dropouts at each stage of the cascade, low proportions of ALHIV achieve viral suppression, which is problematic for current prevention approaches [6,7]. To achieve UNAIDS 90-90-90 targets for adolescents, we need to accurately measure progress in routine “test and treat” settings and implement interventions, which reduce dropouts along the cascade. However, we have limited data on the HIV care cascade among adolescents living in SSA [2,8,9].

The HPTN 071 (PopART) trial was a three-arm community randomized trial in 12 communities in Zambia and nine in South Africa (SA) evaluating the impact of a combination HIV prevention package, including universal HIV testing and treatment, on community-level HIV incidence [10,11]. Within this trial was a substudy called PopART for Youth (P-ART-Y), which evaluated the acceptability and uptake of the PopART HIV prevention package among young people (YP) with a special focus on adolescents aged 10–19 years [12]. It also assessed the need for specific youth-targeted interventions in the context of community-wide universal HIV testing and treatment.

We have previously reported on uptake of testing among adolescents aged 15–19 years from four communities in Zambia during the first 11 months of the P-ART-Y study from October 2015 to September 2016 [12]. Here we report on the HIV care cascade after the addition of youth-targeted interventions for adolescents aged 10–19 years from 14 communities in Zambia and SA, during the period September 2016 to December 2017. We highlight the main successes and gaps in the cascade and disaggregate the data by country, sex, and age.

## Methods

### Trial design and setting

The HPTN 071 (PopART) trial was a three-arm community randomized trial implemented in 21 communities in Zambia and SA [10,11]. The 21 communities were divided into seven matched triplets (four triplets in Zambia and three in SA), within which communities were similar in geography, size, and estimated HIV prevalence at the start of the trial. Communities in each triplet were randomly assigned to one of three arms: Arm A receiving the full PopART intervention including universal HIV testing and ART for people living with HIV regardless of CD4 count, Arm B receiving the full PopART intervention with ART provided according to national guidelines, and Arm C being the standard of care. Between April and October 2016, national guidelines for initiating ART were changed in both countries, to start ART regardless of CD4 count; therefore, the intervention in Arms A and B became identical during this analysis period. Further details of the PopART trial are described elsewhere [10].

The P-ART-Y study had three phases: qualitative baseline studies and collection of process data from the PopART trial (Phase 1); addition of youth-targeted interventions (Phase 2); and a cross-sectional survey to determine the effect of the intervention on knowledge of HIV status among adolescents (Phase 3; Appendix 1).

### The study intervention

The PopART combination HIV prevention package was delivered by trained community health workers called community HIV care providers (CHiPs) via a door-to-door approach, with treatment and care services provided by local government clinics [10]. The CHiPs delivered the intervention over 4 years (November 2013 to December 2017) in three rounds (R1–3) of which they visited all households, offered to explain the intervention, and asked permission to enumerate (list) all household members, providing a count of all individuals in the communities. CHiPs enumerated all household members including those absent, irrespective of age. The P-ART-Y study was implemented during R2 (July 2015 to August 2016) and R3 (September 2016 to December 2017) of the PopART intervention (Appendix 1).

CHiPs also offered HIV counseling and testing services (HTS) to all eligible household members, supported LTC for all people living with HIV, and provided ongoing ART adherence support, condom promotion and provision, screening for tuberculosis and sexually transmitted infections, and referrals to voluntary medical male circumcision for HIV-negative men. They worked in pairs within an allocated zone (consisting of 350–500 households) of a community and arranged repeat household visits to monitor LTC services and offer HTS for those absent at previous visits.

### Informed consent

To take part in the intervention, all household members aged ≥18 years were asked for verbal informed consent, whereas those <18 years were asked for their verbal assent and their parents or guardians (head of household or appropriate responsible adult) for their verbal consent. For child-headed households, informed consent was given by the head of the household. If verbal consent was obtained, the individual was considered a study participant. Written consent for HIV testing was sought in adolescents aged ≥16 years in Zambia and those aged ≥12 years in SA, with parental written consent needed for adolescents below those ages, as per national guidelines.

### Youth-targeted interventions

Youth-targeted interventions were implemented in July 2016 (approximately a year after beginning of R2) and throughout R3 to increase participation, HIV testing uptake, and LTC. These were offered in Arms A and B in addition to the PopART intervention (Appendices 1 and 2). Interventions included the employment of youth counselors, training parents, and clinic staff to enable them to engage better with adolescents and reinforcing HIV prevention school-based activities. Youth-friendly corners received financial and/or technical support so that they could be transformed into hubs where adolescents could be mentored and supported by peers and where educational materials and condoms were distributed.

Adolescents aged 10–14 years were screened for risk of HIV infection using a screening tool based on four questions [13]:

1. Has the child ever been admitted to hospital?
2. Does the child have recurring skin problems?
3. Are one or both parents of the child deceased?
4. Has the child had poor health in the past 3 months?

If the answer was yes to one or more of these questions, the adolescent was classified as “at risk.” Adolescents classified as “at risk” and those aged ≥15 years were prioritized by CHiPs to be tested for HIV.

### Data collection and analysis

We report on adolescents aged 10–19 years from 14 intervention (Arms A and B) communities in Zambia and SA, covering the period September 2016 to December 2017 (R3) of the PopART intervention. The proportions of key indicators (uptake of HIV testing, knowledge of HIV status, ART coverage, time to initiate ART, and retention on ART) are disaggregated by country, sex, and age group. The analysis was limited to R3 because (1) in R1, we did *not* have ethics permission to collect data from those aged <18 years; (2) data had previously been published from four of the Zambian communities in the first year of the P-ART-Y study [14]; and (3) reliable data were not available from SA in the previous rounds [15].

All analyses were repeated separately for Arms A and B to investigate whether any stark differences could help explain the final PopART trial results [15,16]. In the PopART trial, HIV incidence differed quite substantially between Arms A and B, with Arm B having a lower incidence than Arm A (despite Arm A having the slightly more intensive intervention for the early period of the study). In addition, formal statistical testing was not done due to the large sample sizes involved, and small *p* values could be obtained for even marginal differences.

An adolescent was classified as knowing their HIV status before the R3 intervention if they self-reported living with HIV (LHIV) or self-reported to have tested HIV negative in the previous 12 months. After the R3 visit, those who received an HIV test and a result from a CHiP were classified as knowing their HIV status, along with those who had self-reported LHIV. *ART coverage* was measured as the proportion of ALHIV who self-report currently being on ART and also report having not missed their pills in the last 3 days. *ART retention* was defined as the proportion who were currently on ART and report not missing any pills in the last 3 days, among those who report ever having started ART.

Self-reported data were collected on whether they were registered for HIV care, had ever taken ART, were currently on ART, and for those who reported taking ART how many pills they had missed in the previous 3 days. For adolescents who reported they were registered for HIV care, CHiPs asked to see their ART card from which the ART card number and date of ART initiation were recorded.

To estimate the time it took to initiate ART after a referral, the R3 data were combined with the R2 data to provide sufficient follow-up time. Kaplan–Meier plots were used to estimate the median time from first referral to ART initiation, using the date the participant was referred to HIV care by the CHiPs as the start of the observation period. Cox regression was used to assess whether time to ART initiation differed by sex or age group, adjusted for age, sex, and trial arm. Data were censored on the date of the last follow-up visit for those who never started ART, and follow-up data up to December 2017 were used.

The coverage against the first two of the UNAIDS 90-90-90 targets (proportion of ALHIV who knew their status and proportion of those who were on ART) was estimated, with extrapolation to the total adolescent population. Definitions and assumptions required for extrapolating these estimates to the whole adolescent population are described (Appendix 3). A sensitivity analysis was performed to estimate coverage against the 90-90 targets if more conservative assumptions were made (Appendix 4), in particular coverage among adolescents who did not participate in R3. Viral load testing was not routinely done in government clinics in Zambia; therefore, information on viral suppression was *not* collected by CHiPs; the study was designed to be similar in both countries.

### Ethical approval

Ethics approval was obtained from the ethics committees of the University of Zambia, Stellenbosch University, and the London School of Hygiene and Tropical Medicine. Permission to conduct the study was received from the Ministry/Department of Health in Zambia and SA, respectively.

## Results

### Participation and uptake of testing

**Figure 1:**
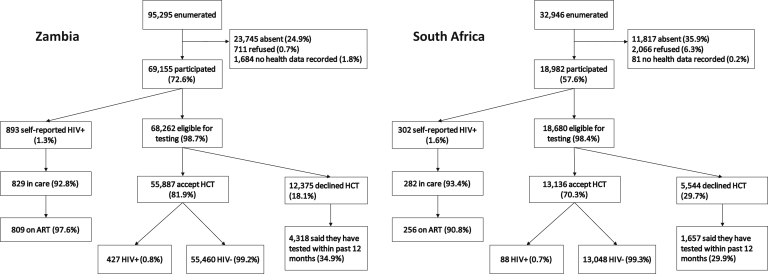
Study intervention participation and HIV testing eligibility and testing uptake among adolescents in Zambia and South Africa.

A total of 128,241 adolescents aged 10–19 years from Zambia and SA were enumerated by the CHiPs in the 14 communities, 95,295 in Zambia and 32,946 in SA (Figure 1). A total of 88,137 adolescents (68.7%) participated in the intervention. Participation was lower in SA, and in both countries, it was lower among boys (Zambia: 75.9% girls vs. 68.8% boys; SA: 60.7% girls vs. 54.2% boys; Table 1). Most adolescents who did not participate were absent; only a small proportion refused to participate or had no health data recorded.

**Table 1:** Adolescents who participated in the intervention, knowledge of HIV status, and ART status among ALHIV

Zambia	Boys			Girls		
	10–14	15–19	Overall	10–14	15–19	Overall
Enumerated	23,218	20,911	44,129	25,830	25,336	51,166
Participated (% among enumerated)	15,429 (66.5%)	14,913 (71.3%)	30,342 (68.8%)	18,281 (70.8%)	20,532 (81.0%)	38,813 (75.9%)
Knows status when first seen in R3 (% among participants)	3,896 (25.3%)	4,961 (33.3%)	8,857 (29.2%)	4,415 (24.2%)	8,425 (41.0%)	12,840 (33.1%)
Accepts testing(% among those eligible)	11,597 (76.3%)	12,888 (87.2%)	24,485 (81.7%)	13,903 (76.9%)	17,499 (86.6%)	31,402 (82.0%)
Tests positive (% among those tested)	53 (.5%)	38 (.3%)	91 (.4%)	69 (.5%)	267 (1.5%)	336 (1.1%)
Knows status after R3 visit (% among participants)	12,788 (82.9%)	13,859 (92.9%)	26,647 (87.8%)	15,242 (83.4%)	19,209 (93.6%)	34,451 (88.8%)
Known positive after R3 visit (% among participants)	276 (1.8%)	179 (1.2%)	455 (1.5%)	273 (1.5%)	592 (2.9%)	865 (2.2%)
On ART when first seen in R3 (% among known positive)	203 (73.6%)	131 (73.2%)	334 (73.4%)	190 (69.6%)	277 (46.8%)	467 (54.0%)
On ART at end of R3 (% among known positive and resident)	210 (86.4%)	133 (80.6%)	343 (84.1%)	189 (85.5%)	315 (70.5%)	504 (75.4%)
South Africa						
Enumerated	8,168	7,399	15,567	8,788	8,591	17,379
Participated (% among enumerated)	4,007 (49.1%)	4,425 (59.8%)	8,432 (54.2%)	4,603 (52.4%)	5,947 (69.2%)	10,550 (60.7%)
Knows status when first seen in R3 (% among participants)	497 (12.4%)	1,295 (29.3%)	1,792 (21.3%)	706 (15.3%)	2,882 (48.5%)	3,588 (34.0%)
Accepts testing (% among those eligible)	2,558 (64.7%)	3,276 (75.0%)	5,834 (70.1%)	2,911 (64.0%)	4,391 (75.6%)	7,302 (70.5%)
Tests positive (% among those tested)	4 (.2%)	11 (.3%)	15 (.3%)	5 (.2%)	68 (1.5%)	73 (1.0%)
Knows status after R3 visit (% among participants)	2,789 (69.6%)	3,729 (84.3%)	6,518 (77.3%)	3,212 (69.8%)	5,365 (90.2%)	8,577 (81.3%)
Known positive after R3 visit (% among participants)	57 (1.4%)	68 (1.5%)	125 (1.5%)	58 (1.3%)	207 (3.5%)	265 (2.5%)
On ART when first seen in R3 (% among known positive)	49 (86.0%)	47 (69.1%)	96 (76.8%)	50 (86.2%)	110 (53.1%)	160 (60.4%)
On ART at end of R3 (% among known positive and resident)	52 (92.9%)	53 (88.3%)	105 (90.5%)	55 (98.2%)	150 (82.9%)	205 (86.5%)

ART = antiretroviral therapy.

Acceptance of HIV testing was higher in Zambia than SA. Among those eligible for testing (i.e., those who did not self-report HIV positive), 81.9% accepted testing in Zambia compared with 70.3% in SA. There was a trend observed with age, with acceptance of testing increasing as age increased, but no difference between sexes or between arms (Appendix 5).

### Knowledge of HIV status

Among participating adolescents, the number who knew their HIV status increased substantially in both arms after R3. In Zambia, 31.4% knew their HIV status before R3 (boys 29.2% and girls 33.1%) compared with 88.3% after R3 (boys 87.8% and girls 88.8%). In SA, the increase was from 28.3% before (boys 21.3% and girls 34.0%) to 79.5% after R3 (boys 77.3% and girls 81.3%; Table 1).

**Figure 2:**
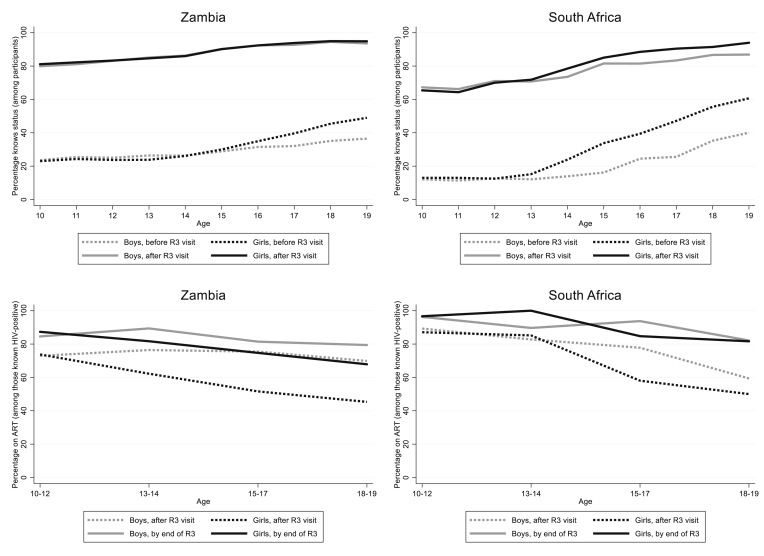
Proportion of adolescents, among participants, who know their HIV status before and at the end of R3 (top two charts) and proportion of known HIV-positive adolescent participants on ART (bottom two charts) stratified by sex and age. *∗Denominator for lower two plots are those known HIV + after the R3 annual visit (so includes newly diagnosed individuals)*.

In both countries before R3, knowledge of HIV status was greater among older adolescents, particularly among girls, but after the intervention, there was little difference between boys and girls (Figure 2). Knowledge of HIV status increased during R3 for all age groups.

Overall, there were 1,710 known ALHIV after R3 (580 boys and 1,130 girls), of whom 1,195 (69.9%) were aware of their status and had disclosed this, whereas 515 (30.1%) were new diagnoses (32.3% in Zambia and 22.6% in SA). The proportion of boys known to be HIV positive among all participants was 1.5% in both countries; among girls, the proportion was 2.2% in Zambia and 2.5% in SA (Table 1). Of the 515 new diagnoses, 335 (65.0%) were girls aged 15–19 years. A higher proportion of 10- to 14-year-old known ALHIV self-reported their HIV status (80.3%) compared with 15- to 19-year-olds (63.3%).

### ART coverage and time to initiate ART

ART coverage before R3 among self-reported ALHIV was 90.6% in Zambia and 84.8% in SA. Overall, ART coverage among known ALHIV (either self-reported or tested positive with CHiPs) was 61.3% in Zambia and 65.6% in SA before R3. By the end of R3, ART coverage had increased among known ALHIV up to 78.7% in Zambia and 87.8% in SA. ART coverage among ALHIV who self-reported their HIV positive status increased from 90.6% to 92.8% in Zambia and from 84.8% to 93.6% in SA. Of the participants newly diagnosed as HIV positive, 44% reported being on ART in Zambia and 65% in SA at the end of R3.

In Zambia, overall ART coverage increased in boys by over 10% and in girls by over 20%. In SA, the increases were larger; in boys, the increase was around 14%, and in girls, it was 26% (Table 1). In both sexes, the largest gains were seen in the older age groups (Figure 2, lower two plots). At the end of R3, the overall percent on ART was greater for boys than girls by almost 9% in Zambia and 4% in SA.

The median time to ART initiation was approximately 5 months in both countries (Appendix 6). There was no evidence to suggest a difference in time to initiate ART between sexes (Zambia, *p* = .25; SA, *p* = .30) or between younger and older adolescents (Zambia, *p* = .11; SA, *p* = .68).

### Retention on ART

Self-reported ART retention was high in all age and sex groups. Retention in Zambia was marginally higher at 98.2% in boys and 94.3% in girls. In SA, retention was 92.2% in boys and 96.3% in girls.

### HIV treatment and care cascade

**Figure 3:**
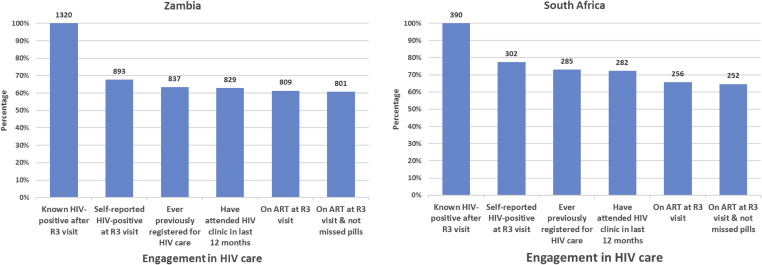
HIV care cascade before Round 3 visit.

The cascade immediately before R3 by country is shown (Figure 3). In both countries, the greatest gap, before intervention, was the first step in the diagnosis of HIV infection. At each step of the cascade, the proportions are lower for Zambia, compared with SA.

### Reaching the 90-90-90 targets

**Figure 4:**
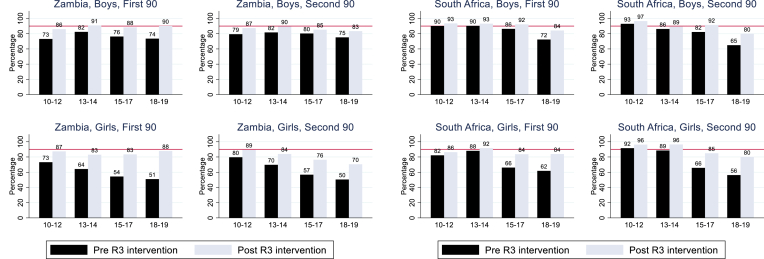
90-90: Estimated knowledge among adolescents of HIV-positive status and ART coverage before and after the R3 intervention with extrapolation to the population, by age group. ∗Denominator for the second 90 are those known HIV + *after* the R3 annual visit (so includes newly diagnosed individuals).

At the end of R3, the first 90 target was met or nearly met in most age groups except girls aged 13–17 years in Zambia and those aged 15–19 years in SA where 83% and 84% knew their HIV status, respectively (Figure 4).

In both countries, the second 90 was achieved or nearly achieved in adolescents aged 10–14 years. In older adolescents in Zambia, the second 90 was not achieved, particularly in girls, with only 76% of the 15- to 17-year-olds and 70% of the 18- to 19-year-olds on ART. In SA, the largest gap was among 18- to 19-year-olds with an estimated 80% on ART in both boys and girls.

Sensitivity analyses were performed, and using our most conservative set of assumptions, estimates of coverage against the first 90 target were 75%–84% for boys and 75%–83% for girls and against the second 90 target were 73%–86% for boys and 68%–86% for girls (Appendix 7).

### Comparing Arms A and B

Most indicators were similar between the two intervention arms; however, some small differences were observed in participation and HIV status in SA. Participation in SA was slightly higher in Arm A compared with Arm B, particularly in girls (girls 5% higher and boys 2.2% higher). The proportion of girls aged 15–19 years with a positive HIV diagnosis was higher in Arm A than in Arm B in SA (3.8% vs. 3.2%). Overall, although time to ART initiation was different by age and sex in Arm A, there was no evidence of a difference between these groups in Arm B. ART coverage was different between arms before R3, but by the end of intervention, most of the difference between arms had disappeared (Appendix 8).

## Discussion

The P-ART-Y study has highlighted progress and key gaps in delivering home-based HTS for adolescents aged 10–19 years in SSA. We present data disaggregated by sex and age, which are relevant from a regional perspective to enable focused and targeted programmatic responses [17]. By breaking down the HIV care cascade by age and sex, we highlight which adolescent subpopulations require specific interventions and attention at each stage. We also demonstrate how far we have come in terms of achieving the first and second UNAIDS 90-90-90 targets and what gaps remain.

We identified low knowledge of HIV status among adolescents before the intervention. However, we see the substantial impact of community-based HTS on increasing this, with more than 80% ALHIV knowing their status in both countries after the intervention, consistent with previous findings [18]. It also resulted in boys and girls having similar levels of knowledge of HIV status.

Knowledge of HIV status was considerably higher than in other studies in SSA [19] because home-based HTS had been offered to adolescents since October 2015. However, approximately one third of adolescents were offered the intervention for the first time during this period because of high mobility and migration in these communities. Historically, HIV programs have struggled to persuade adolescents to periodically test for HIV as they often underestimate their risk [20]. The results from adolescent surveys in SSA conducted between 2011 and 2016 showed that only 10% of boys and 12% of girls had tested for HIV in the last 12 months [21].

Overall, the proportion of known ALHIV was comparatively low, highlighting a window of opportunity to vigorously scale up HIV prevention efforts [22]. In Zambia, for example, approximately 60% of YP aged 15–24 years lack correct knowledge about HIV transmission and prevention, emphasizing the urgent need to increase awareness [23]. Prevention services, such as pre-exposure prophylaxis should be linked to HIV testing to maximize the additional benefit of promoting uptake [24]. For adolescent girls and young women, tailored interventions such as the DREAMS initiative have been shown to be effective [25].

Although most adolescents were HIV negative, we saw a sharp increase in infection among 17- to 19-year-old girls. A high proportion of the lifetime risk of acquiring HIV occurs in young women is due to a complex interplay of biology, gender-power disparities, and socioeconomic and other social triggers affecting vulnerability in women [26,27]. In SSA, adolescent girls aged 15–19 years are two to eight times more likely than boys of the same age to become HIV infected [28]. Whether age-disparate sexual partnering between older men and adolescent girls or young women is important for HIV transmission in SSA is still unclear [29].

The intervention came close to achieving both the first and second 90-90-90 targets for younger boys. Gaps in the first 90 were seen in girls aged 13–17 and 15–19 years in Zambia and SA, respectively. Recent data from 16 SSA countries indicate several gaps in HIV testing coverage, particularly among adolescents [30]. Population-based HIV impact assessments conducted in Malawi, Zambia, and Zimbabwe found that only 46% of youths (aged 15–24 years) LHIV were aware of their HIV status, compared with 65% of 25- to 34-year-olds and 78% of 35- to 59-year-olds [31].

To achieve the 90-90-90 targets among adolescents, the main emphasis needs to be placed on HIV testing and subsequent LTC [32]. Recent evidence from Zambia suggests that the greatest gap among YP aged 15–25 years is the first 90 in line with our findings [23]. Several promising approaches such as HIV self-testing, same-day ART initiation, differentiated models of care, point of care CD4 count, facilitated linkage strategies, and improved clinic services have resulted in improved outcomes along the cascade [[32], [33], [34]].

Time to initiate ART was the same as for adults in the same population [35]. However, other studies have shown worse pre-ART and ART outcomes among adolescents compared with adults due to a range of factors resulting in delayed ART initiation [36,37]. We saw gaps in the second 90 among older adolescents aged 15–19 years in both countries. Data from SSA show poor ART initiation and retention among ALHIV [38]. In the SEARCH study, only 64% of previously diagnosed youth LHIV were on ART at baseline, compared with 81% of older adults [38]. In this study, we put in place several strategies to encourage LTC. These included CHiPs escorting clients to the clinic; use of specially trained counselors; working with existing community health care workers to track clients; following up with clients who missed clinic appointments; and holding meetings with clinic staff to review which CHiP clients had, or had not, linked to care and/or started ART.

Implementing the “treat all” strategy in SSA requires dedicated efforts to address the unique needs of adolescents to improve outcomes along the HIV care cascade. Although provider-initiated testing plays an important role in increasing HTS uptake, it is not sufficient on its own. Community, home, and school-based approaches that recognize the unique needs of adolescents are required to address the problem of undiagnosed HIV infection among adolescents [39]. Compared with other testing strategies, home-based HTS offers many advantages and has been effective in enhancing testing uptake [40].

Our study had some limitations. The purpose of this study was to report on HIV care cascade outcomes before and after the delivery of a single round of the intervention, using data collected as part of service delivery. However, given the possibility of secular trends affecting uptake of HIV testing and ART, it limits the strength of our conclusion. In addition, there was no PopART intervention in the control arm, so there was no CHiPs intervention data collected in Arm C. However, to overcome this, at the end of R3, a cross-sectional survey was conducted in Arm C to provide comparative data; results have been reported elsewhere (article under review). Although small differences were observed between the two intervention trial arms, these were minor, and the picture in the two arms was broadly similar.

Although it was a limitation that ART uptake, ART retention, and prior HIV testing were self-reported, by the end of the intervention, CHiPs had established good relationships and trust with their clients and had received thorough and ongoing training on electronic data collection, giving overall confidence in self-reported data. Also, specifically for ART uptake, individuals who reported being on ART were asked to produce their clinic ART card as verification.

Information on viral suppression was not collected by CHiPs as part of the intervention service delivery, as viral load testing was not routinely done in Zambia.

We were not able to reach all enumerated adolescents, especially in SA because of high rates of absenteeism. It was challenging to find older boys at home because of their greater involvement in income generation activities. These harder-to-reach groups are not only absent from homes but do not frequently attend clinics and may require alternative approaches to engage them in HTS.

Finally, we also cannot disentangle the effects of the main PopART intervention and the additional P-ART-Y targeted interventions from the data. There was no control group of 10- to 19-year-olds who only received a “standard” PopART intervention, allowing us to disentangle the specific effect of the youth-targeted strategies.

## Conclusions

The first two UNAIDS 90-90-90 targets were markedly increased among adolescents after the delivery of a PopART package of services, which included adolescent-specific interventions. No opportunity to provide HIV prevention approaches to the large majority of HIV-negative adolescents should be missed.

## Supplementary Data

Appendices 1–8
