# Supplementary material for: HIV Care Cascade Among Adolescents in a “Test and Treat” Community-Based Intervention: HPTN 071 (PopART) for Youth Study
Source: J Adolesc Health. 2021 Apr;68(4):719–27. doi: 10.1016/j.jadohealth.2020.07.029 (PMC8022105; doi:10.1016/j.jadohealth.2020.07.029)
Supplement: Appendices 1–8 [file mmc1.docx]

**Appendix 1: P-ART-Y study implementation phases and Timelines**

**
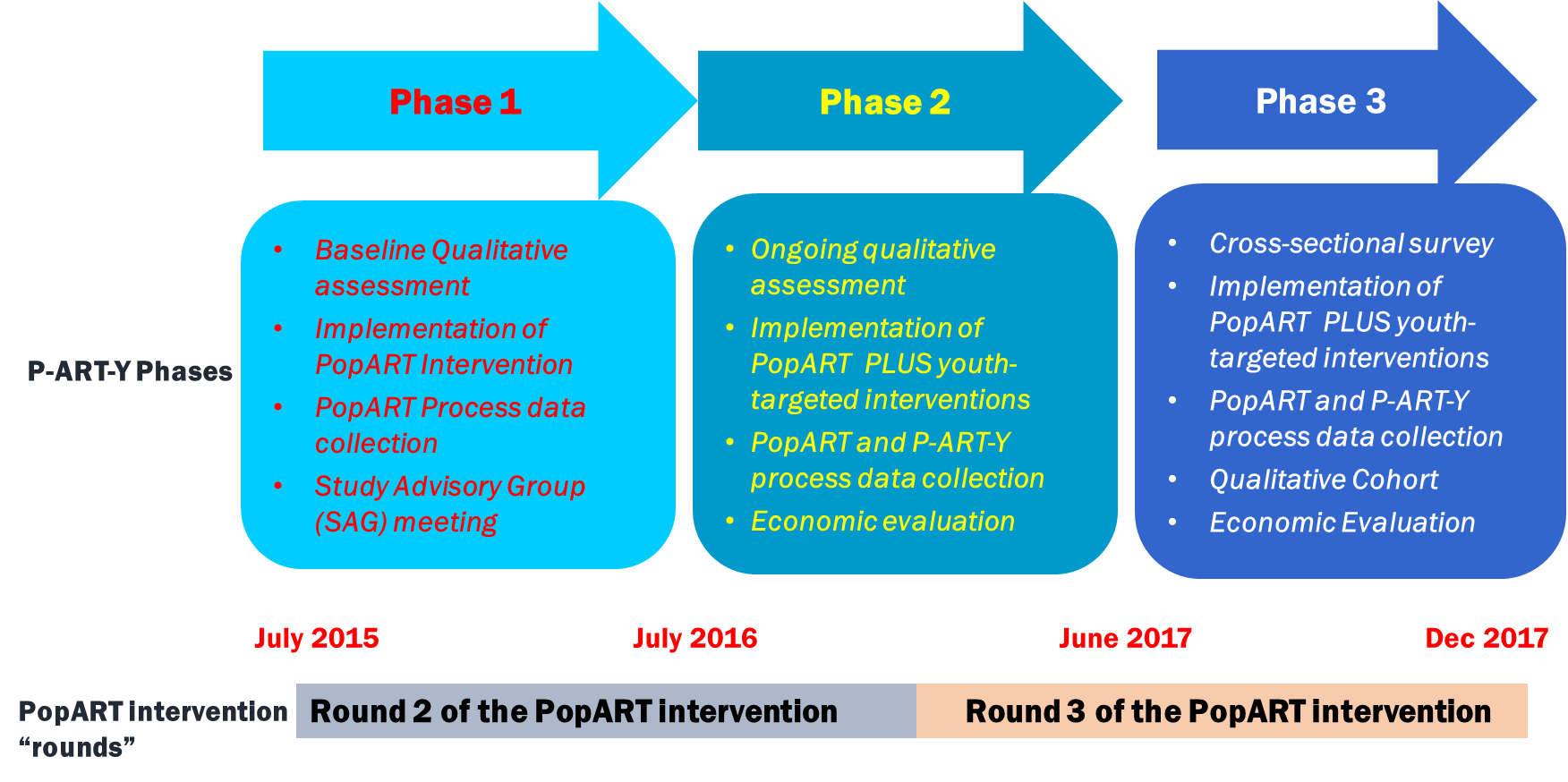
**

*The data collection “rounds” were periods in which the CHiPs collected data. The three rounds were: Round 1 (R1): November 2013 –June 2015; Round 2 (R2): July 2015 – August 2016; and Round 3 (R3): September 2016 – December 2017. Each data collection round lasted approximately 15 to 20 months.*

**Appendix 2: Detailed description of P-ART-Y Youth Targeted interventions**

P-ART-Y Youth Targeted Interventions were delivered by CHiPs, trained Youth focused counsellors, peer educators and mentors. Exact activities were defined for each community. The intervention was made up of the following components:

*1.The Values Clarification training* was intended to support clinic health providers, CHIPs and Parents with the necessary training, capacity-building, and ongoing mentorship of practical skills needed to engage with young people. The training content was an adaptation of the WHO content guidance on delivering HIV services for young people. The training supported efforts to build strong positive relationships with adolescents and improve communication with them; provided knowledge on how to encourage parents to raise sexually healthy children; to understand concepts around adolescent development; promoted the role of parents in delivering the sexual risk prevention messages to adolescents; provided knowledge, skills, comfort and confidence needed to engage with adolescents appropriately about sexual risk reduction; provided tools that aimed at assisting communication/dialogue.

2. *Use of youth counsellors, trained peer educators and mentors*

Youth counsellors were selected on a competitive basis following a public advertisement. The requirements were as follows: aged between 19-30 years, GCE ‘O’ level graduates who had experience in adolescent counselling, preferably adolescents living with HIV, experience in implementing community-based youth HIV prevention, treatment and care activities, and be residents of the communities. Their role was to support adolescent counselling, recommend specific training needs for the CHiPs on adolescent counselling, mentor CHiPs on how to handle challenging cases and to help with counselling at Youth Friendly Corners (YFC) and will work hand in hand with the Focal point persons at YFC

3. *Clinic Youth Friendly Corners activities*

Youth friendly corners received financial and/or technical support so that they could be transformed into hubs where adolescents could be mentored and supported by peers, and where educational materials and condoms were distributed.

The youth-friendly corners were improved by renovating the existing spaces designed for youth at the clinic, providing IEC material and educational books, providing HIV prevention education and services including HIV testing and condom distribution; providing incentives for clinic based staff to continue being involved in youth friendly corner activities. Additionally, clinic youth friendly corners, were used for promotion of the PopART interventions and as a fast-tract linkage for youth who needed to access clinic services or track those who have not linked to care via phone/sms.

*4. P-ART-Y Community ‘Safe’ Spaces*

‘Community Safe Spaces’, an HPTN071 (PopART) community based initiative to optimise PopART intervention strategies for young people was an intervention targeted at young people aged 13-24 years. These events were intended to re-imagine the interaction between young people and CHiPs. Since most of the PopART intervention activities were delivered door to door, ‘Safe spaces’ responded to community based- door-door service delivery inadequacies and these events were envisaged to attract young people and engage in a dialogue with CHiPs. The objectives were to: (i) equip CHiPs with the skills and confidence to engage with young people (ii) provide an alternative platform for CHiPs to engage with young people and discuss uncomfortable and often not talked about issues outside the home environment (iv) engaging and eliciting opinions from young people who are clients of the PopART intervention, and (iii) identifying HIV related service needs

*5. Develop adolescent-focussed messages targeted for parents, young people, health care providers and the community as a whole*

Formed the basis of health information given to adolescents and young people during health events, school and community interventions.

The messaging and communication around P-ART-Y focussed on providing basic information about the study as well as disseminating core messages during the course of outreach activities. These messages were presented through digital and print media in the form of a website/Facebook page and flyers/leaflets that were distributed at outreach events. The primary objective of the messaging was to sensitise young people and community members to the P-ART-Y study, to enable them to deliver adolescent appropriate, sensitive, and demand-driven health services

*6. School based activities·*

In schools (primary and secondary government schools) the information on HIV prevention, HIV risks; safer sex behaviors and other topics was given by peer mentors and educators through the use of standardized curriculum and participatory innovative interactive tools (like the use of video, “join-in circuit” and grassroots soccer approach). In country Ministry of Education guidelines on Comprehensive Sexual Education and HIV prevention activities in schools were implemented depending on the need in the particular school. The information was tailored for youth and delivered via various platforms such as games, quizzes, debates, mobile health promotions and health education/ talks/ peer education.

*7. Youth Health Campaigns*

Adolescents not in school were reached through youth health campaigns or youth health days conducted quarterly by the study team in collaboration with other community based youth organizations. Different types of community activities were conducted including Quiz, Door to door, sensitization, Drama and dance, one on one discussions, Sport, Adolescent community meetings, use of role models. The intervention package was offered at these campaigns with emphasis on linkage to care at the clinic for those who were HIV positive. Promotion of the PopART intervention package was also conducted.

**Appendix 3: Definitions of Indicators and UNAIDS 90-90-90 Assumptions**

The “**first 90 *before the intervention*”** defines adolescents who knew their HIV-positive status before the CHiPs’ R3 visit, among those estimated to be LHIV at the time of this visit. The **“first 90 *after the intervention*”** is measured with the same denominator, but is measured immediately after the CHiPs’ visit and includes the impact of HIV testing in R3. The **“second 90 *before the intervention*”** is the proportion of ALHIV who are currently on ART among those known to be HIV-positive just after CHiPs’ R3, whereas the **“second 90 *after the intervention*”** is defined as the proportion on ART at the final CHiP follow up visit.

In order to make estimates of the first and second 90 extrapolated to be representative of the population, we made the following assumptions (with stratification on sex, community, and age group):

1. Among adolescents who participated in R3, all who were LHIV and who knew their HIV-positive status, self-reported this to the CHiPs
2. The proportion of ALHIV who knew their HIV-positive status was the same in those who did, and did not, participate in the intervention, *immediately prior to R3*
3. ART uptake among non-participants in R3 was the same as among participants *immediately prior the R3 visit*
4. HIV prevalence was the same among adolescents who did, and did not, participate in R3

**Appendix 4: Assumptions for sensitivity analysis for UNAIDS 90-90-90 estimates**

The assumptions for the sensitivity analysis, to give more conservative estimates (stratified by age/sex/community groups) were:

1. Participants who did not self-report being HIV positive and declined a test were assumed to have double the HIV prevalence of those who accepted a test.
2. Of those who did not test but were HIV-positive, the proportion who knew their status immediately prior to R3 was assumed to be 50% of the proportion in the rest of the HIV-positive participants. And of those, it was assumed that the proportion on ART at the start of Round 3 was 50% of the value among participants who self-reported being HIV-positive.
3. Among non-participants the assumed HIV prevalence was 25% higher than in participants
4. Among HIV-positive non-participants it was assumed that knowledge of status prior to Round 3 was 20% lower than knowledge among HIV-positive participants
5. Among HIV-positive non-participants who knew their HIV status, ART coverage was assumed to be 20% lower than among participants who self-reported HIV-positive in Round 3.

**Appendix 5: Uptake of HIV testing across ages, stratified by sex and arm**

**Appendix 6: Time to event graphs by country**

**
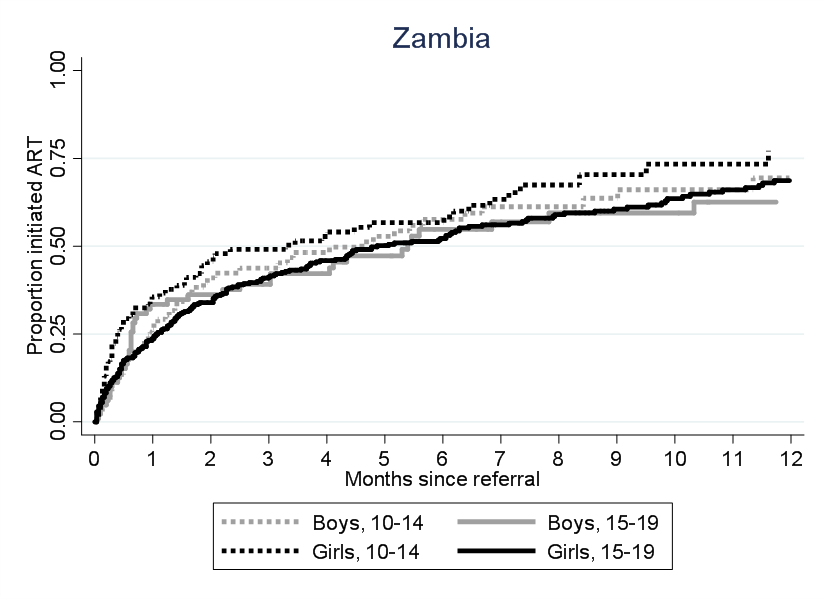
**

**Appendix 7: First and Second 90: Sensitivity analysis graphs for pooled data**


**Appendix 8: Adolescents enumerated, participated in the intervention and knowledge of HIV status before and after annual round and ART status among ALHIV in Zambia and SA. (Arm A compared to Arm B)**

|  | **Arm A** | | | | | |  | **Arm B** | | | | | |
| --- | --- | --- | --- | --- | --- | --- | --- | --- | --- | --- | --- | --- | --- |
|  | **Boys** | | | **Girls** | | |  | **Boys** | | | **Girls** | | |
| **Zambia** | **10 to 14** | **15 to 19** | **Overall** | **10 to 14** | **15 to 19** | **Overall** |  | **10 to 14** | **15 to 19** | **Overall** | **10 to 14** | **15 to 19** | **Overall** |
| Enumerated | 11377 | 10295 | 21672 | 12628 | 12063 | 24691 |  | 11841 | 10616 | 22457 | 13202 | 13273 | 26475 |
| Participated  (% among enumerated) | 7604 (66.8%) | 7672 (74.5%) | 15276 (70.5%) | 8947 (70.9%) | 9795 (81.2%) | 18742 (75.9%) |  | 7825 (66.1%) | 7241 (68.2%) | 15066 (67.1%) | 9334 (70.7%) | 10737 (80.9%) | 20071 (75.8%) |
| Knows status when first seen in R3 (% among participants) | 1858 (24.4%) | 2324 (30.3%) | 4182 (27.4%) | 2049 (22.9%) | 3853 (39.3%) | 5902 (31.5%) |  | 2038 (26.0%) | 2637 (36.4%) | 4675 (31.0%) | 2366 (25.3%) | 4572 (42.6%) | 6938 (34.6%) |
| Accepts testing (% among those eligible) | 5600 (74.8%) | 6783 (89.1%) | 12383 (82.0%) | 6623 (74.8%) | 8473 (87.8%) | 15096 (81.6%) |  | 5997 (77.7%) | 6105 (85.3%) | 12102 (81.3%) | 7280 (79.0%) | 9026 (85.5%) | 16306 (82.4%) |
| Tests positive (% among those tested) | 21  (0.4%) | 16  (0.2%) | 37  (0.3%) | 25  (0.4%) | 12  (1.5%) | 152  (1.0%) |  | 32  (0.5%) | 22  (0.4%) | 54  (0.4%) | 44  (0.6%) | 140  (1.6%) | 184  (1.1%) |
| Knows status after R3 visit (% among participants) | 6222 (81.8%) | 7194 (93.8%) | 13416 (87.8%) | 7291 (81.5%) | 9210 (94.0%) | 16501 (88.0%) |  | 6566 (83.9%) | 6665 (92.0%) | 13231 (87.8%) | 7951 (85.2%) | 9999 (93.1%) | 17950 (89.4%) |
| Known positive after R3 visit (% among participants) | 140  (1.8%) | 75  (1.0%) | 215  (1.4%) | 113  (1.3%) | 277  (2.8%) | 390  (2.1%) |  | 136  (1.7%) | 104  (1.4%) | 240  (1.6%) | 160  (1.7%) | 315  (2.9%) | 475  (2.4%) |
| On ART when first seen in R3 (% among known positive) | 108 (77.1%) | 55  (73.3%) | 163 (75.8%) | 82  (72.6%) | 129 (46.6%) | 211 (54.1%) |  | 95  (69.9%) | 76  (73.1%) | 171 (71.3%) | 108 (67.5%) | 148 (47.0%) | 256 (53.9%) |
| On ART at end of R3 (% among known positive & resident) | 111 (86.7%) | 55  (80.9%) | 166 (84.7%) | 86  (88.7%) | 147 (70.3%) | 233 (76.1%) |  | 99  (86.1%) | 78  (80.4%) | 177 (83.5%) | 103 (83.1%) | 168 (70.6%) | 271 (74.9%) |
| **South Africa** |  |  |  |  |  |  |  |  |  |  |  |  |  |
| Enumerated | 3800 | 3556 | 7356 | 4154 | 4175 | 8329 |  | 4368 | 3843 | 8211 | 4634 | 4416 | 9050 |
| Participated (% among enumerated) | 1903 (50.1%) | 2165 (60.9%) | 4068 (55.3%) | 2298 (55.3%) | 2968 (71.1%) | 5266 (63.2%) |  | 2104 (48.2%) | 2260 (58.8%) | 4364 (53.1%) | 2305 (49.7%) | 2979 (67.5%) | 5284 (58.4%) |
| Knows status when first seen in R3 (% among participants) | 249 (13.1%) | 649 (30.0%) | 898 (22.1%) | 372 (16.2%) | 1491 (50.2%) | 1863 (35.4%) |  | 248 (11.8%) | 646 (28.6%) | 894 (20.5%) | 334 (14.5%) | 1391 (46.7%) | 1725 (32.6%) |
| Accepts testing (% among those eligible) | 1223 (65.1%) | 1621 (75.7%) | 2844 (70.8%) | 1452 (64.0%) | 2180 (75.5%) | 3632 (70.4%) |  | 1335 (64.3%) | 1655 (74.3%) | 2990 (69.5%) | 1459 (64.0%) | 2211 (75.7%) | 3670 (70.6%) |
| Tests positive (% among those tested) | 2  (0.2%) | 5  (0.3%) | 7  (0.2%) | 4  (0.3%) | 32  (1.5%) | 36  (1.0%) |  | 2  (0.1%) | 6  (0.4%) | 8  (0.3%) | 1  (0.1%) | 36  (1.6%) | 37  (1.0%) |
| Knows status after R3 visit (% among participants) | 1351 (71.0%) | 1854 (85.6%) | 3205 (78.8%) | 1636 (71.2%) | 2712 (91.4%) | 4348 (82.6%) |  | 1438 (68.3%) | 1875 (83.0%) | 3313 (75.9%) | 1576 (68.4%) | 2653 (89.1%) | 4229 (80.0%) |
| Known positive after R3 visit (% among participants) | 27  (1.4%) | 29  (1.3%) | 56  (1.4%) | 32  (1.4%) | 113  (3.8%) | 145  (2.8%) |  | 30  (1.4%) | 39  (1.7%) | 69  (1.6%) | 26  (1.1%) | 94  (3.2%) | 120  (2.3%) |
| On ART when first seen in R3 (% among known positive) | 23  (85.2%) | 21  (72.4%) | 44  (78.6%) | 27  (84.4%) | 70  (61.9%) | 97  (66.9%) |  | 26  (86.7%) | 25  (64.1%) | 51  (73.9%) | 23  (88.5%) | 37  (39.4%) | 60  (50.0%) |
| On ART at end of R3 (% among known positive & resident) | 23  (88.5%) | 23  (85.2%) | 46  (86.8%) | 30  (96.8%) | 88  (88.0%) | 118 (90.1%) |  | 29  (96.7%) | 30  (90.9%) | 59  (93.7%) | 25  (100%) | 62  (76.5%) | 87  (82.1%) |
